# New Departure in Dentistry

**Published:** 1878-09-20

**Authors:** 


### NEW DEPARTURE IN DENTISTRY.

Certain prominent writers in Destistry advocate what is called the "New Departure," as to the best material for filling teeth, affirming that the old theory that gold is the best material is incorrect, and that plastic fillings are the best. Gold, instead of being placed at the head of the list, is now at the bottom, as follows:

First, gutta percha; second, amalgum; third, tin; fourth, oxychloride of zinc; fifth, gold. Dr. Parsons, an experienced dentist of Savannah, writing on this subject, says: "That gold, by reason of its conductivity, is antagonistic to a healthy condition of the electro-magnetic constitution of the teeth.

